# The impact of spray-induced gene silencing on cereal phyllosphere microbiota

**DOI:** 10.1186/s40793-024-00660-8

**Published:** 2025-01-08

**Authors:** Poorva Sundararajan, Samrat Ghosh, Bekele Gelena Kelbessa, Stephen C. Whisson, Mukesh Dubey, Aakash Chawade, Ramesh Raju Vetukuri

**Affiliations:** 1 Department of Plant Breeding, Swedish University of Agricultural Sciences, Alnarp, Sweden; 2 Cell and Molecular Sciences, The James Hutton Institute, Invergowrie, Dundee, UK; 3 Department of Forest Mycology and Plant Pathology, Swedish University of Agricultural Sciences, Uppsala, Sweden

**Keywords:** Microbiome, Amplicon sequence variants (ASVs), *Fusarium graminearum*, Spray induced gene silencing (SIGS), Double-stranded RNA (dsRNA), Phyllosphere, Wheat, Barley

## Abstract

**Background:**

Fusarium head blight (FHB) is a major disease affecting cereal crops including wheat, barley, rye, oats and maize. Its predominant causal agent is the ascomycete fungus *Fusarium graminearum*, which infects the spikes and thereby reduces grain yield and quality. The frequency and severity of FHB epidemics has increased in recent years, threatening global food security. Spray-induced gene silencing (SIGS) is an alternative technique for tackling this devastating disease through foliar spraying with exogenous double-stranded RNA (dsRNA) to silence specific pathogen genes via RNA interference. This has the advantage of avoiding transgenic approaches, but several aspects of the technology require further development to make it a viable field-level management tool. One such existing knowledge gap is how dsRNA spraying affects the microbiota of the host plants.

**Results:**

We found that the diversity, structure and composition of the bacterial microbiota are subject to changes depending on dsRNA targeted and host studied, while the fungal microbiota in the phyllosphere remained relatively unchanged upon spraying with dsRNA. Analyses of fungal co-occurrence patterns also showed that *F. graminearum* established itself among the fungal communities through negative interactions with neighbouring fungi. Through these analyses, we have also found bacterial and fungal genera ubiquitous in the phyllosphere, irrespective of dsRNA treatment. These results suggest that although rarer and less abundant microbial species change upon dsRNA spray, the ubiquitous bacterial and fungal components of the phyllosphere in wheat and barley remain unchanged.

**Conclusion:**

We show for the first time the effects of exogenous dsRNA spraying on bacterial and fungal communities in the wheat and barley phyllospheres using a high-throughput amplicon sequencing approach. The results obtained further validate the safety and target-specificity of SIGS and emphasize its potential as an environmentally friendly option for managing Fusarium head blight in wheat and barley.

**Supplementary Information:**

The online version contains supplementary material available at 10.1186/s40793-024-00660-8.

## Background

Wheat (*Triticum* spp.) and barley (*Hordeum vulgare*) are major cereal crops grown for food and feed worldwide [1, 2]. In 2022, 154 million tonnes of barley and 808 million tonnes of wheat were produced around the world, underscoring their importance as primary crops [3]. Unfortunately, their production is hampered by several diseases and pests [4] including Fusarium head blight (FHB). It is mainly caused by the ascomycete fungus *Fusarium graminearum* Schwabe [5], which grows best in warm and humid or semi-humid regions [6, 7]. FHB is one of the most destructive fungal crop diseases and causes billions of dollars of losses of wheat and barley [8–11]. In addition to yield losses, FHB-causing fungi promote the accumulation of toxic secondary fungal metabolites (mycotoxins) such as deoxynivalenol (DON), nivalenol (NIV), and zearalenone (ZEA) that significantly reduce grain quality [12, 13]. The mycotoxin DON is most frequently detected in food wheat and barley, and is harmful to human, animal, and ecosystem health [14–16]. Due to ongoing global climate change and changes in cropping systems, the frequency and severity of FHB epidemics have increased in recent years, posing challenges to human food security, animal nutrition, and the international grain trade [5, 17, 18].

Several disease control strategies have been used to mitigate the increasing threat of FHB and mycotoxin accumulation in grains, including cultural practices, biological control [19], induction of host resistance [5, 20], precision genome editing with CRISPR/Cas9 [21], and foliar spraying with fungicides [22]. RNAi-based strategies such as host-induced gene silencing (HIGS) have been reported to reduce crop losses caused by fungi, oomycetes, nematodes, and insect pests [23–27]. However, because HIGS involves the host-expression of hairpin RNAs (hpRNAs) or small RNAs (sRNAs) targeting genes in the interacting pathogen, its practical utility is limited by several factors including the limited transformability of various crops and the poor acceptance of genetically modified (GM) crops by many consumers [28]. These problems motivated the development of an alternative strategy that requires no genetic modification: spray-induced gene silencing, or SIGS [29]. This strategy involves spraying leaves with double-stranded RNAs (dsRNA) or sRNAs to specifically silence selected pathogen genes. The potential of SIGS as a tool for managing fungal and oomycete diseases and insect pests has been successfully demonstrated through several studies [26, 28, 30–36]. For example, one study showed that *F. graminearum* can take up exogenous dsRNA and that spraying detached barley leaves with dsRNA targeting the *F. graminearum CYP51A*, *CYP51B*, and *CYP51C* genes reduced the incidence and severity of infection [37]. Another study showed that using SIGS to target *TRI6*, a transcription factor involved in DON biosynthesis in *F. graminearum*, reduced FHB infection and DON levels in wheat heads inoculated under greenhouse conditions [38]. A third study demonstrated that targeting key components of the fungal RNAi machinery with SIGS reduced barley infection by *F. graminearum* [39]. SIGS has thus shown great potential for minimizing crop losses caused by filamentous pathogens.

Despite these promising results, to turn it into a practical disease management strategy, several facets of SIGS still need to be understood. Besides the disease reduction, the broader effects of spraying dsRNA on the host, such as the effect on the phyllosphere microbiome have received little attention. The phyllosphere (aerial habitat) is influenced by the plant and houses an intricate, dynamic and heterogeneous microbial community consisting primarily of bacteria, filamentous fungi, yeasts, algae and protozoans [40, 41]. The diversity and composition of these microbial communities are also sensitive to several factors that interact over space and time, including crop protection measures (e.g., pesticide treatment), synthetic fertilizers, environmental factors and host genotypes [42–45]. Conversely, several studies have also illustrated that microbial communities can enhance the host-plant’s growth, health, and tolerance to abiotic and biotic stresses. This is achieved through various mechanisms, including secretion of growth-promoting phytohormones, enhancement of nutrient availability, secretion of secondary metabolites that are toxic to pathogenic microbes, and induction of systemic acquired resistance [46–49]. Therefore, it is imperative to ascertain changes to the host microbiome when developing new plant protection approaches. This can be done by exploiting recent advances in high-throughput sequencing and other meta-omic techniques that have facilitated the profiling of microbial communities and their functions in various crops, including wheat and barley [50, 51]. In this study, we sought to assess if dsRNA affects the microbial communities of the phyllosphere and how its effects on these interactions change upon *F. graminearum* infection. Our initial hypothesis was that dsRNA would not significantly alter the phyllosphere microbial communities in wheat and barley. To test this hypothesis, we used high-throughput amplicon sequencing techniques to characterize the diversity, structure and composition of the phyllosphere microbiota before and after spraying plants with dsRNA. For this purpose, two *F. graminearum* genes that are essential for FHB disease progression and are targeted by fungicides were utilized to synthesize dsRNA: cytochrome P450 lanosterol C-14α-demethylase (*FgCyp51A*, *FgCyp51B* and *FgCyp51C*) [52] and succinate dehydrogenase B subunit (*FgSdhB*) [53]. We also assessed the effects of *F. graminearum* inoculation on phyllosphere microbial composition after dsRNA spraying by comparing diversity metrics for the microbial communities of non-inoculated and inoculated plants.

## Materials and methods

### Plant and fungal material

Seeds of the spring wheat breeding line SW141580 (Lantmännen) and spring barley market cultivar Tellus were germinated in Petri dishes lined with damp Whatman filter paper to induce uniform germination. The germinated seedlings were transplanted into 9 × 9 × 8 cm pots filled with well-draining soil and grown under controlled climatic conditions with 16 h of 200 µmol/m^2^/s daylight and 8 h of darkness, and day/night temperatures of 22/21°C. *F. graminearum* PH-1 was grown on potato dextrose agar (PDA) (VWR International) in Petri dishes and incubated at 19 °C for seven days. Carboxymethyl cellulose (CMC) media (7.5 g of carboxymethyl cellulose, 0.5 g of yeast extract, 0.25 g of MgSO_4_.7H_2_0, 0.5 g of NH_4_NO_3_ and 0.5 g of KH_2_PO_4_ dissolved in 1 l of distilled water) was used for conidiation. Agar plugs from seven-day-old PDA cultures were used to inoculate CMC media. The inoculated CMC media was incubated at 28 °C for seven days with constant shaking and illumination to produce conidia. They were then collected by passing the culture through two layers of cheesecloth followed by centrifugation to remove media, and subsequently resuspending in sterile water. The concentration of conidia was calculated using a Fuchs-Rosenthal chamber and adjusted to 20,000 conidia/ml for plant infection.

### In-vitro dsRNA synthesis

RNA was extracted from mycelia collected from seven-day-old PDA plates using the RNeasy Plant Mini kit (Qiagen). First-strand synthesis was then carried out using the iScript cDNA Synthesis kit (Bio-Rad) with one microgram of the extracted RNA as the template. Primers containing the T7 promoter sequence were designed for the *FgCyp51A* (FGSG_04092), *FgCyp51B* (FGSG_01000), *FgCyp51C* (FGSG_11024) and *FgSdhB* (FGSG_05610) gene sequences using NCBI Primer-BLAST (Table 1) [54]. Polymerase chain reaction was performed using Phusion polymerase (ThermoFisher Scientific) with *F. graminearum* cDNA as the template and the dsRNA-specific T7 primers, and following the reaction conditions recommended by the manufacturer. The PCR product was purified using the QIAquick PCR Purification kit (Qiagen) before proceeding with in-vitro transcription. Double-stranded RNA was synthesized using the MEGAscript RNAi Kit (ThermoFisher Scientific) and the appropriate PCR-amplified products as templates. In addition, the control template provided with the kit was used to synthesize non-specific dsRNA to serve as a control in subsequent experiments. The control template consisted of a linearized TRIPLEscript plasmid containing the 1.85 kb Xenopus elongation factor 1α gene under the transcriptional control of tandem SP6, T7, and T3 promoters. Gel electrophoresis was performed in a 1% agarose gel to confirm synthesis of appropriate dsRNA products. The concentration of the purified dsRNA was measured using a nano-drop spectrophotometer.

### Plant assay – dsRNA treatment, *F. graminearum* infection and sample collection

The *FgCyp51* dsRNA was obtained by mixing equal concentrations of the individually synthesized *FgCyp51A*,* FgCyp51B* and *FgCyp51C* dsRNAs. Four-week-old spring wheat and barley plants were sprayed with *FgCyp51*/*FgSdhB* dsRNA (10 µg of dsRNA per plant) using an airbrush and compressor (CoCraft and Biltema, respectively). Untreated plants and plants sprayed with 10 µg of non-specific dsRNA were included as experimental controls. Twenty-four hours after spraying, half the plants from each treatment were drop inoculated with 20 µl of 20,000 *F. graminearum* conidia/ml. Four biological replicates were established for each treatment. Leaf samples were collected four days after spraying using three punches from a 1.5 ml microcentrifuge tube each measuring 10.8 mm diameter and stored at -80 °C.

### DNA extraction

DNA was extracted from the collected leaf samples using a modified protocol of the DNeasy PowerSoil Pro kit (Qiagen) as mentioned below. The frozen leaf samples were ground to a powder in a pre-chilled mortar and pestle filled with liquid nitrogen, before proceeding with the recommended protocol from the manufacturer. The washing step using solution EA was repeated three times to ensure the removal of phenolic compounds.

### Amplicon sequencing

Extracted DNA was sent for Amplicon sequencing (LGC Genomics). The 799F-1115R primer pair targeting the 16 S rRNA gene and the ITS1Kyo2F-ITS86R primer pair targeting the ITS gene sequence were used for bacterial and fungal amplification [55, 56], respectively. The 799F and ITS1Kyo2F primers used in this study are discriminating primers and avoid amplification of host DNA during sequencing [57, 58]. In total, 126 samples were used for amplicon sequencing. Of these, 64 samples were from wheat (32 for bacterial amplification and 32 for fungal amplification) and 62 were from barley (with 31 samples for bacteria and 31 for fungi). Each treatment consisted of four biological replicates, except the non-specific dsRNA (Nsp) treatment in barley, where only three replicates were included due to poor DNA quality leading to no amplification. The PCR reactions were performed with 1–10 ng of DNA extract in a total volume of 1 µl, 15 pmol of the appropriate forward and reverse primers (Table 1) in a 20 µL volume of 1 x MyTaq buffer containing 1.5 units MyTaq DNA polymerase (Bioline GmbH, Luckenwalde, Germany), and 2 µl of BioStabII PCR Enhancer (Sigma-Aldrich Co.). All forward and reverse primers contained the same 10-nt barcode sequence. PCRs were performed for 30–40 cycles (30–33 cycles for samples amplified with 799F-1115R and 35–40 cycles for samples amplified with ITS1Kyo2F-ITS86R) using the following parameters: pre-denaturation at 96 °C for 1 min, denaturation at 96 °C for 15 s, annealing at 55 °C for 30 s and extension at 70 °C for 90 s. No template reactions were included as negative controls during PCR as part of standard procedure at LGC Genomics.

The DNA concentration of the amplicons was assessed by gel electrophoresis. In these experiments, amplicon pools representing up to 48 samples were created by mixing roughly 20 ng of amplicon DNA from each sample, each of which carried a unique barcode. The amplicon pools were purified by using one volume of Agencourt AMPure XP beads (Beckman Coulter, Inc., IN, USA) to remove primer dimers and other small mispriming products, and further purification was performed with MiniElute columns (QIAGEN GmbH, Hilden, Germany). The purified amplicon pool DNA (100 ng each) was used to construct Illumina libraries using the Ovation Rapid DR Multiplex System 1–96 (NuGEN Technologies, Inc., CA, USA). Illumina libraries (Illumina, Inc., CA, USA) were pooled and size selected by preparative gel electrophoresis. Sequencing was done on an Illumina MiSeq using V3 Chemistry.

**Table 1:** Primers used for in-vitro transcription of dsRNA and amplicon sequencing

Primer type	Primer Name	Primer Sequence
In-vitro dsRNA synthesis	T7 Cyp51A FW	GTAATACGACTCACTATAGGGCGGTCCATTGACAATCCCCG
	T7 Cyp51A RV	GTAATACGACTCACTATAGGGGCAGCAAACTCGGCAGTGAG
	T7 Cyp51B FW	GTAATACGACTCACTATAGGGCAGCAAGTTTGACGAGTCCC
	T7 Cyp51B RV	GTAATACGACTCACTATAGGGAGAGTTCATAAGGTGCTTCA
	T7 Cyp51C FW	GTAATACGACTCACTATAGGGATTGGAAGCACCGTACAATA
	T7 Cyp51C RV	GTAATACGACTCACTATAGGGCATTGGAGCAGTCATAAACA
	T7 Fg SdhB FW	GTAATACGACTCACTATAGGGGGACCTTGTCCCTGATCTGA
	T7 Fg SdhB RV	GTAATACGACTCACTATAGGGGCTTCTTGATCTCGGCAATC
Amplicon sequencing	Bac_799F	AACMGGATTAGATACCCKG
	Bac_1115R	AGGGTTGCGCTCGTTG
	Fun_ITS1Kyo2F	TAGAGGAAGTAAAAGTCGTAA
	Fun_ITS86R	TTCAAAGATTCGATGATTCA

### Processing of amplicon data

Raw Illumina paired-end reads were demultiplexed with Sabre2 [59] and adapters were trimmed with the bbduk.sh script [60]. Next, demultiplexed and adapter trimmed data were imported into the QIIME2-2022.8 pipeline [61]. Primers were trimmed with cutadapt plugin of QIIME2, and the demux plugin was used for quality checking. The DADA2 [62] plugin of QIIME2 was used for quality trimming, dereplication, chimera removal and generation of amplicon sequence variants (ASVs). The QIIME2-compatible SILVA v138.9 [63] and UNITEv9 [64] databases were used for bacterial and fungal taxonomy annotation, respectively. Three standard output files obtained from the DADA2 plugin (the count table, fasta file and assigned taxonomy data) and one external sample metadata file were merged into a phyloseq object using the R package “phyloseq v1.44” [65]. Before generating the phyloseq object, unassigned ASVs and ASVs assigned to the chloroplasts and mitochondria were filtered out.

### Statistical analysis

After generating the phyloseq object, all statistical analyses were performed in R v 4.2.0 [66]. Data from the phyloseq object were first rarefied using the lowest sequencing depth (wheat − 16173 and 11398 reads per sample for bacteria and fungi, respectively; barley − 12663 and 4961 reads per sample for bacteria and fungi, respectively). Package UpSetR v1.4.0 [67] was used for generating UpSet plots. For core-microbiome analysis the microbiome package [68] was used. Normality of the data was checked using the shapiro.test() function of the stats v3.6.2 package. The alpha diversity metric Shannon index (H) and the statistical significance test (one-way ANOVA followed by pairwise t-test) were computed with the vegan v 2.6-4 package [69]. Beta diversity was evaluated using Bray-Curtis distance-based principal coordinate analysis (PCoA). Statistical significance (PERMANOVA) for distance matrices was computed using the adonis() function of the vegan v 2.6-4 package [69], while significance for pairwise comparisons was calculated using the pairwise.adonis() function of the pairwiseAdonis v 0.4 package [70]. Simultaneously, rarefied data were normalized to obtain relative abundance values (%), and taxon compositions based on these values were plotted using the plot_bar() function. The R package phylosmith [71] was used for microbial network analysis. Network construction was done using the Spearman rank correlation method with the p-value and rho cut-off set at 0.05 and 0.8, respectively. Network topology was calculated using the igraph v 1.5.1 package [72] and a customised script was used for the ZiPi plots. Based on standard criteria, all ASVs were categorized into four groups: peripherals (Zi < 2.5 and Pi < 0.62), connectors (Pi > 0.62), module hubs (Zi > 2.5) and network hubs (Zi > 2.5 and Pi > 0.62). The package microbiomeMarker v 1.6.0 [73] was used for linear discriminatory analysis effect size computation (LefSe) [74] with a linear discriminatory score (LDA) and p-value cut-off of 3.0 and < 0.05, respectively.

## Results

### High-throughput amplicon sequencing characterizes bacterial and fungal communities

**Fig. 1:**
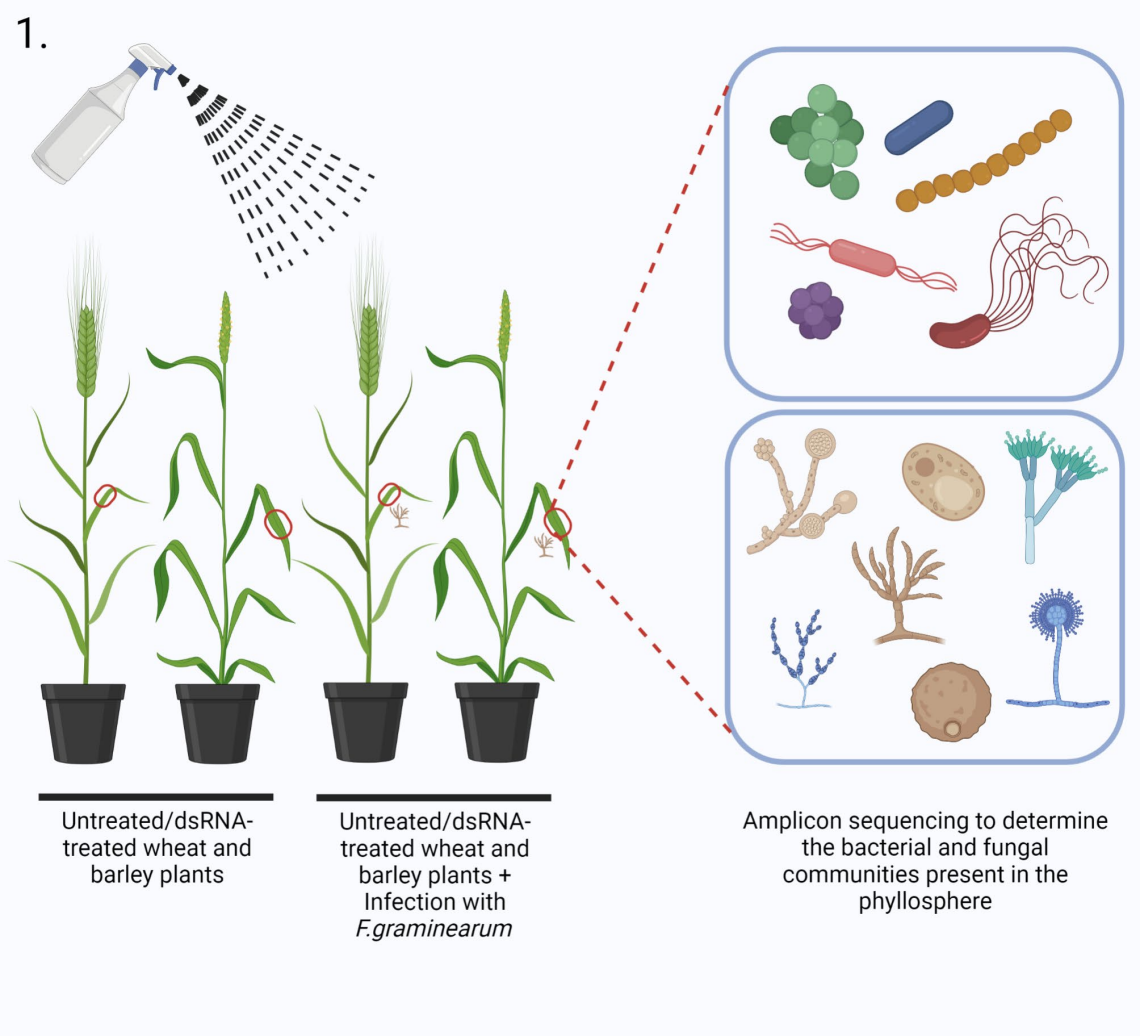
Schematic depiction of the experimental set-up used to study the effects of dsRNA spraying on the phyllosphere microbiota. Created with BioRender.com

**Fig. 2:**
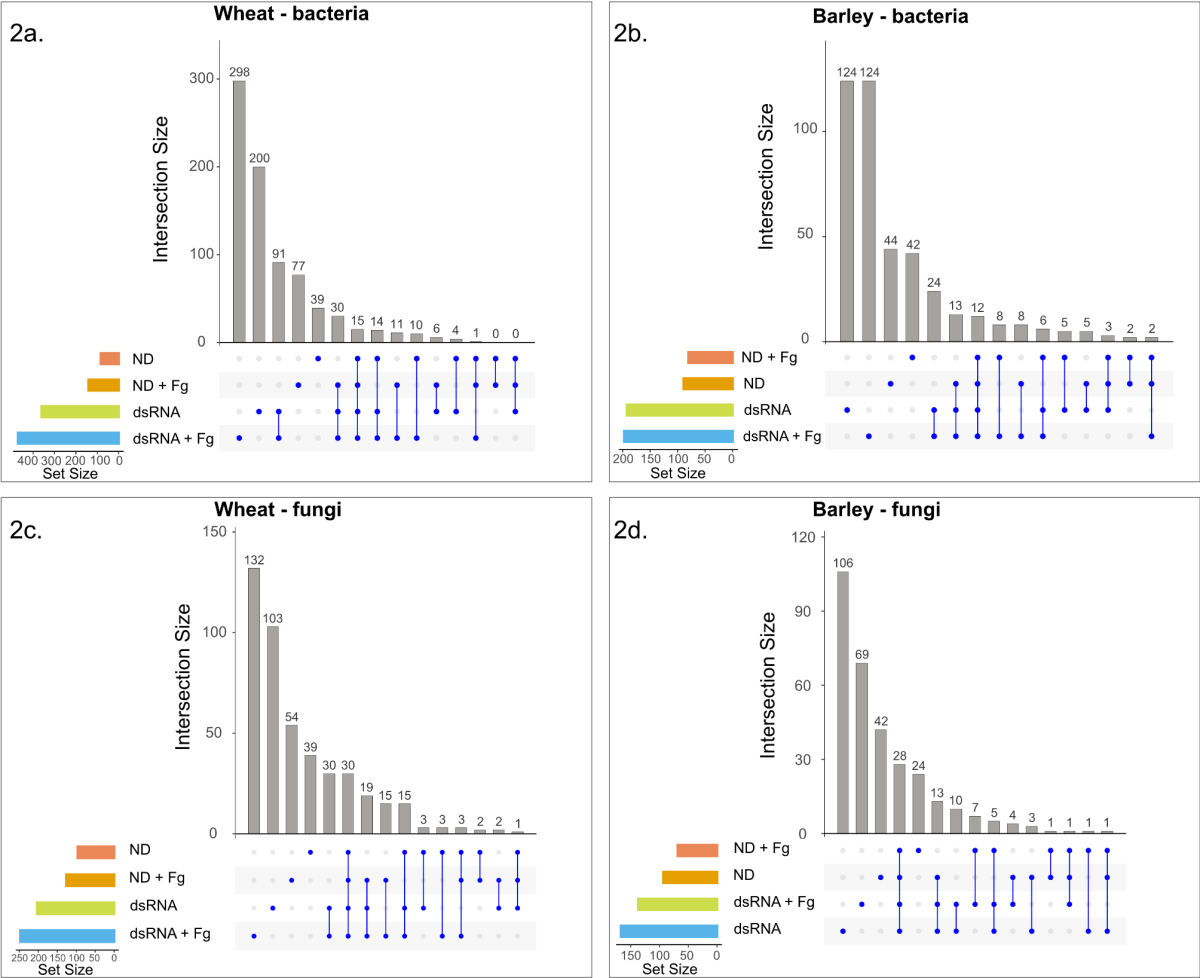
UpSet plots showing the set sizes of the different treatment groups (ND, ND + Fg, dsRNA, dsRNA + Fg), and the shared and unique bacterial (**a** and **b**) and fungal (**c** and **d**) ASVs identified in wheat and barley, respectively. The blue dots represent the individual sets of the different treatment groups and the blue lines represent the intersecting sets

Figure 1 depicts the experimental set-up used in this study. Samples from each host consisted of eight different control and dsRNA treatments - no dsRNA (ND), no dsRNA + Fg (ND + Fg), non-specific dsRNA (Nsp), non-specific dsRNA + Fg (Nsp + Fg), dsRNA Cyp51 (Cyp51), dsRNA Cyp51 + Fg (Cyp51 + Fg), dsRNA SdhB (SdhB), dsRNA SdhB + Fg (SdhB + Fg). Amplicon sequencing produced a total of 1,896,748 bacterial and 1,898,726 fungal filtered reads from wheat and 1,953,614 bacterial and 2,334,856 fungal filtered reads from barley, respectively (Additional file 1: Table S1). A plateau was observed in the rarefaction curves of all the sequenced samples, indicating that the samples provided adequate diversity and coverage for the tested conditions (Additional file 2: Fig. S1). A total of 1018 bacterial and 460 fungal ASVs were obtained from wheat as well as 548 bacterial and 333 fungal ASVs from barley (Additional file 1: Table S1; Additional file 3: Table S2; Additional file 4: Table S3). The proportion of artefactual bacterial reads (ASVs) was 1018/1252 (filtered/unfiltered) in wheat and 548/943 (filtered/unfiltered) in barley. No artefactual fungal reads were found in both barley & wheat. To elucidate the changes induced by the dsRNA spray treatments, the eight treatments were further grouped into four treatment groups: no dsRNA (ND), no dsRNA + Fg (ND + Fg), dsRNA (Nsp, Cyp51 and SdhB), and dsRNA + Fg (Nsp + Fg, Cyp51 + Fg and SdhB + Fg). Details of the shared and unique ASVs found in the different treatment groups are shown in Fig. 2a-d, Additional file 5: Table S4 and Additional file 6: Table S5. For both bacterial and fungal ASVs and across both hosts, a higher number of unique ASVs than common ASVs were found in most of the treatment groups, with most of the unique ASVs being found in lower abundance or belonging to rarer taxa. This indicates that both dsRNA spray and *F. graminearum* inoculation selectively affect rare taxa. Fusarium abundance across the different treatments was quantified by plotting the number of reads that correspond to the genus Fusarium in the eight different treatments (Additional file 2: Fig. S2).

### The diversity of phyllosphere microbial communities after double-stranded RNA spraying

**Fig. 3:**
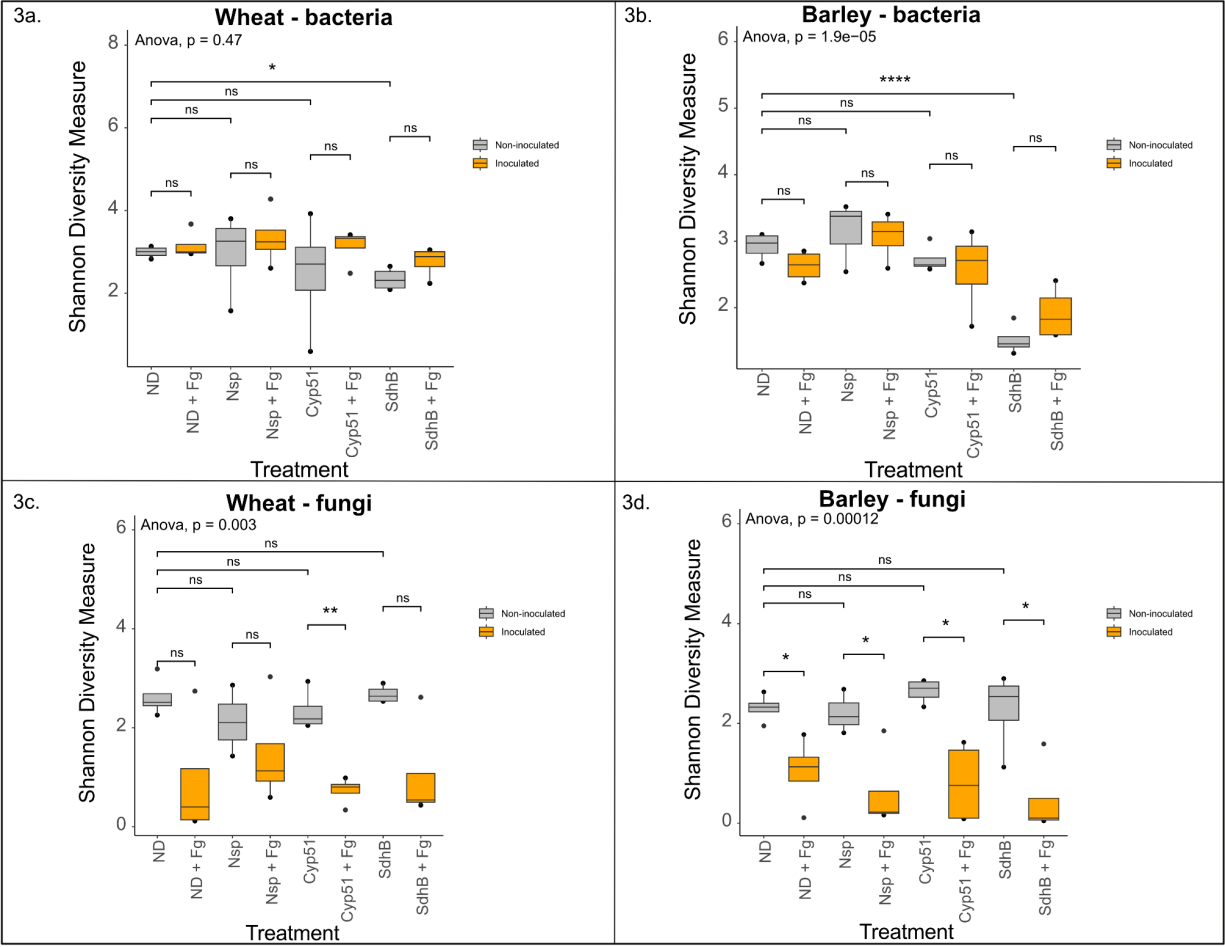
Visualization of alpha diversity measures. Box plots show the Shannon alpha diversity metrics for bacterial (**a** and **b**) and fungal (**c** and **d**) communities in wheat and barley, respectively. The non-inoculated treatments (ND, Nsp, Cyp51 and SdhB) are marked in grey and the *F. graminearum* inoculated treatments (ND + Fg, Nsp + Fg, Cyp51 + Fg, SdhB + Fg) are marked in orange. Statistical significance was determined using one-way ANOVA followed by pairwise t-tests

The alpha diversity measures of the bacterial and fungal taxa were plotted for all eight treatments using the Shannon diversity index (Fig. 3a-d) (Additional file 7: Table S6). Similar bacterial taxonomic evenness was observed across all the treatments in both wheat and barley (Fig. 3a, b), except the SdhB-sprayed samples that showed significantly lower evenness in barley. Further pairwise comparisons revealed the diversity of the dsRNA SdhB-sprayed samples to be significantly different from the no dsRNA (ND) samples in both hosts (pairwise t-test, *p* < 0.05). In both wheat and barley, the fungal taxonomic evenness remained similar between the native state in ND and the dsRNA treatments (Nsp, Cyp51 and SdhB) (Fig. 3c, d), indicating that the stability of the fungal communities is maintained upon dsRNA spray. However, irrespective of the treatment used, inoculation with *F. graminearum* resulted in lower fungal taxonomic evenness (ND + Fg, Nsp + Fg, Cyp51 + Fg, SdhB + Fg) in both hosts. In particular, significant differences were observed upon pairwise comparisons of the following: Cyp51 vs. Cyp51 + Fg in wheat (Fig. 3c), and ND vs. ND + Fg, Nsp vs. Nsp + Fg, Cyp51 vs. Cyp51 + Fg and SdhB vs. SdhB + Fg in barley (Fig. 3d) (pairwise t-test, *p* < 0.05) (Additional file 7: Table S6).

**Fig. 4:**
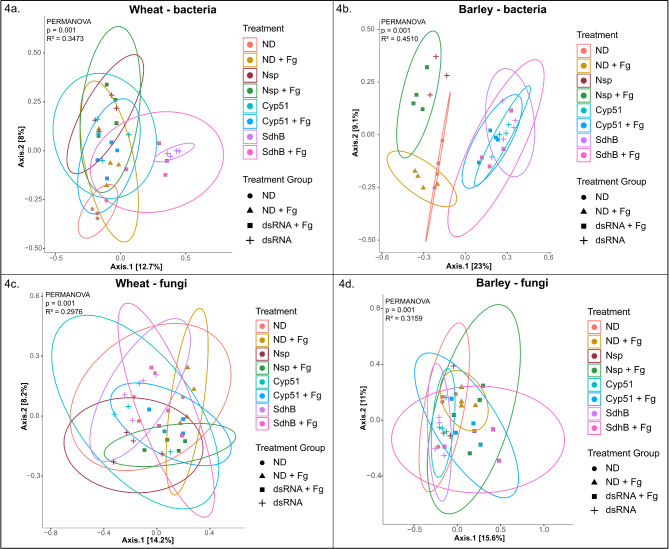
Visualization of beta diversity using principal coordinate analysis (PCoA) plots based on Bray-Curtis distances for bacterial (**a** and **b**) and fungal (**c** and **d**) samples in wheat and barley, respectively. The colours distinguish the eight different treatments, while the shapes distinguish the treatment groups. The confidence level = 0.95 of different treatments are denoted by the confidence ellipsoids. Statistical significance was determined by PERMANOVA/Adonis (number of permutations: 999)

Principal coordinate analyses (PCoA) were performed using the Bray-Curtis dissimilarity and ordination plots based on the first two principal coordinates (PCs) were created to visualize differences and similarities in microbial community diversity between the treatments (Fig. 4a-d). In wheat, partial differentiation of the ND, SdhB and SdhB + Fg bacterial communities was observed, while a considerable overlap was observed between the rest of the treatments (Fig. 4a). This showed that the bacterial composition of the ND, SdhB and SdhB + Fg treatments varied from the bacterial composition of the rest of the treatments. In barley, clear clustering of the bacterial communities between the different treatment groups was observed (Fig. 4b). There was also a noticeable overlap in the bacterial community structure of the targeted-dsRNA samples (Cyp51, Cyp51 + Fg, SdhB and SdhB + Fg). This pointed to dissimilarities in the composition of bacterial communities between the control and targeted-dsRNA treatments. These observations were consistent with a permutational multivariate analyses of variance (PERMANOVA/Adonis; Number of permutations = 999) of the Bray–Curtis distance matrix. The overall Adonis values for the wheat and barley bacterial communities were *p* < 0.001, R2 = 0.3473 and *p* < 0.001, R2 = 0.4510, respectively. However, pairwise comparisons of individual treatments revealed no significant differences (Pairwise Adonis test, *p* > 0.05) (Additional file 8: Table S7). Partial clustering of the fungal communities between the non-inoculated and inoculated samples was observed in both wheat and barley, indicating dsRNA spray resulted in fungal community structures similar to the hosts’ native state, but *F. graminearum* inoculation caused shifts in the community structure (Fig. 4c, d). The overall Adonis values were *p* < 0.001, R2 = 0.2976 for wheat and *p* < 0.001, R2 = 0.3159 for barley. Pairwise comparisons of individual treatments also revealed no significant differences (Pairwise Adonis test, *p* > 0.05) (Additional file 8: Table S7).

### Composition of the phyllosphere microbial communities before and after dsRNA spraying

**Fig. 5:**
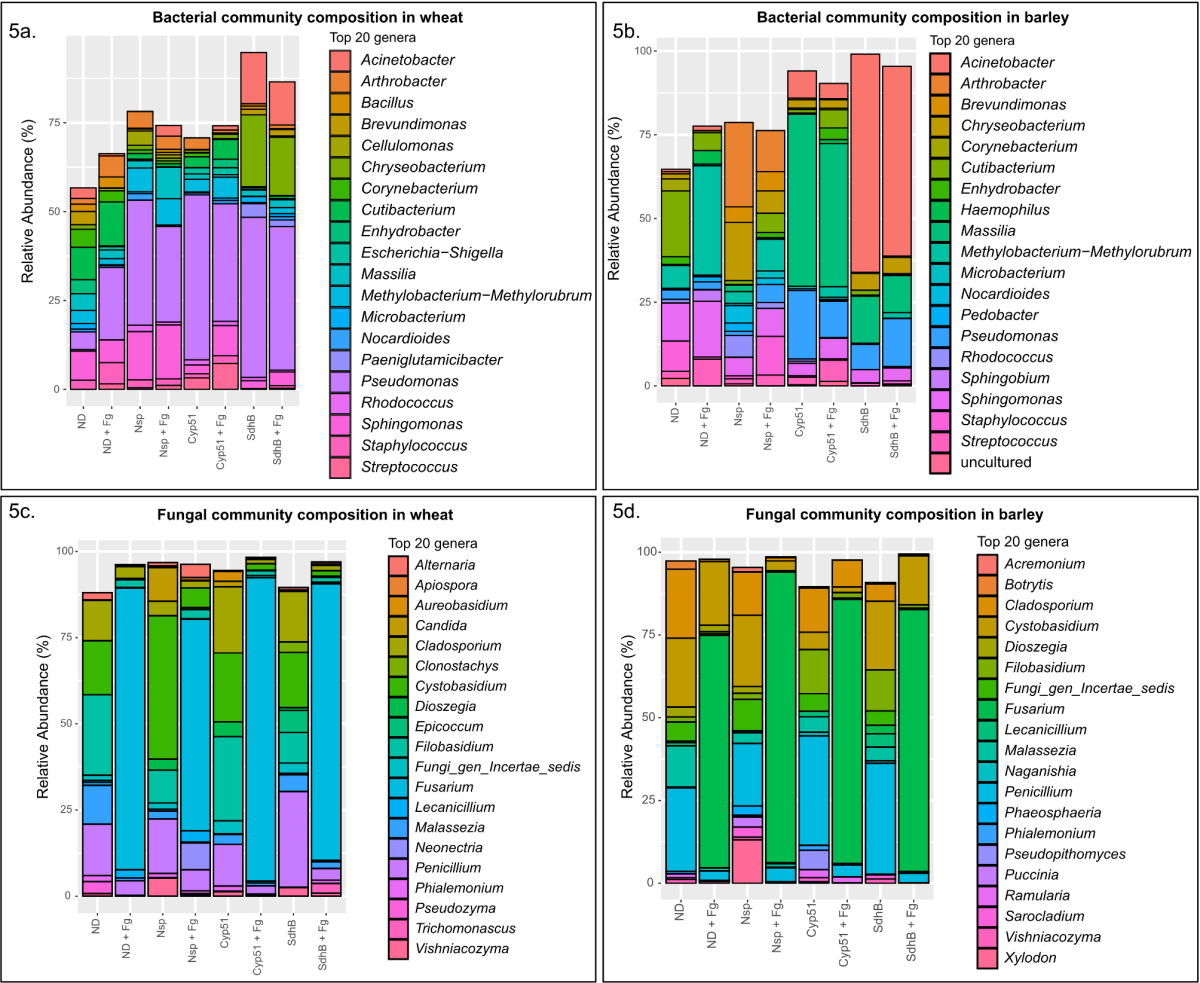
Microbial community composition plots at the genus level. The relative abundance of the top 20 bacterial (**a** and **b**) and fungal (**c** and **d**) genera identified in different treatments in wheat and barley, respectively

The relative abundance of the ASVs present in the different treatments and treatment groups was plotted to characterize the composition of the bacterial and fungal communities (Additional file 9: Table S8 and Additional file 10: Table S9). At the phylum level, the bacterial communities in both hosts were dominated by Proteobacteria (wheat: 46 -73%, barley: 29 − 91%) and Actinobacteria (wheat: 9 − 34%, barley: 2 − 50%) (Additional file 2: Fig. S3a, b). The fungal communities were dominated by Ascomycota (wheat: 35 − 95%, barley: 43 − 95%) and Basidiomycota (wheat: 5 − 63%, barley: 4 − 47%) (Additional file 2: Fig. S3c, d). The relative abundances of the top 20 bacterial and fungal ASVs at the genus level are shown in Fig. 5a-d.

*Pseudomonas* was the most abundant genus among the wheat bacterial communities for all treatments except the no-dsRNA treatment (ND). Compared to the plant’s native state in ND, the relative abundance of *Pseudomonas* increased upon both dsRNA spray and *F. graminearum* inoculation. Other genera ubiquitous across all treatments in wheat include *Sphingomonas*, *Cutibacterium*, *Corynebacterium*, *Staphylococcus*, *Arthrobacter*, *Massilia*, *Methylobacterium-Methylorubrum*, *Brevundimonas*, *Microbacterium* and *Nocardioides*. In addition, an increase in the relative abundance of *Acinetobacter* and *Chryseobacterium* was observed in the dsRNA SdhB samples (SdhB, SdhB + Fg) (Fig. 5a, Additional file 2: Fig. S4a). In barley, while the genera that were most abundant remained roughly the same across treatments, differences in the relative abundance of individual genera were observed. The bacterial genera *Pseudomonas*, *Sphingomonas*, *Staphylococcus*,* Massilia*, *Cutibacterium*, *Methylobacterium-Methylorubrum* and *Streptococcus* were present in all the treatments. *Acinetobacter* predominated in SdhB and SdhB + Fg samples, while *Massilia* was in high relative abundance in Cyp51 and Cyp51 + Fg samples (Fig. 5b, Additional file 2: Fig. S4b). In both no dsRNA (ND) and non-specific dsRNA (Nsp) samples, *F. graminearum* inoculation shifted the relative abundance of bacterial communities, while this was not observed in targeted dsRNA samples.

Among the fungal communities identified in wheat, the genera *Cladosporium*, *Cystobasidium*, *Filobasidium* and *Penicillium* were relatively abundant in all treatments, whereas *Lecanicillium*, *Apiospora*, *Fungi_gen_Incertae_sedis*, *Phialemonium*, *Vishniacozyma* and *Malassezia* were ubiquitous across all treatments in varying amounts (Fig. 5c, Additional file 2: Fig. S4c). Similar to wheat, the genera *Cladosporium*, *Cystobasidium*, *Filobasidium* and *Penicillium* were relatively abundant across all treatments in barley as well, while *Lecanicillium*, *Fungi_gen_Incertae_sedis*, *Vishniacozyma* and *Malassezia* were ubiquitous across all treatments but in varying amounts (Fig. 5d, Additional file 2: Fig. S4d). In both hosts, we observed a significant shift upon *F. graminearum* inoculation in the relative abundances of ascomycetes and basidiomycetes across the treatment groups (Additional file 2: Fig. S3c, d). Additionally, the genus *Fusarium* dominated the samples inoculated with *F. graminearum* in both hosts, showing a clear change in composition of the fungal communities upon pathogen inoculation and colonization. However, no obvious changes in composition were observed between the no dsRNA (ND) and dsRNA (Nsp, Cyp51, SdhB) samples or between their corresponding inoculated treatments (ND + Fg, Nsp + Fg, Cyp51 + Fg, SdhB + Fg), indicating a change in fungal composition only upon *F. graminearum* inoculation.

### Discriminatory analysis reveals taxa that shape the phyllosphere microbiota in wheat and barley

**Fig. 6:**
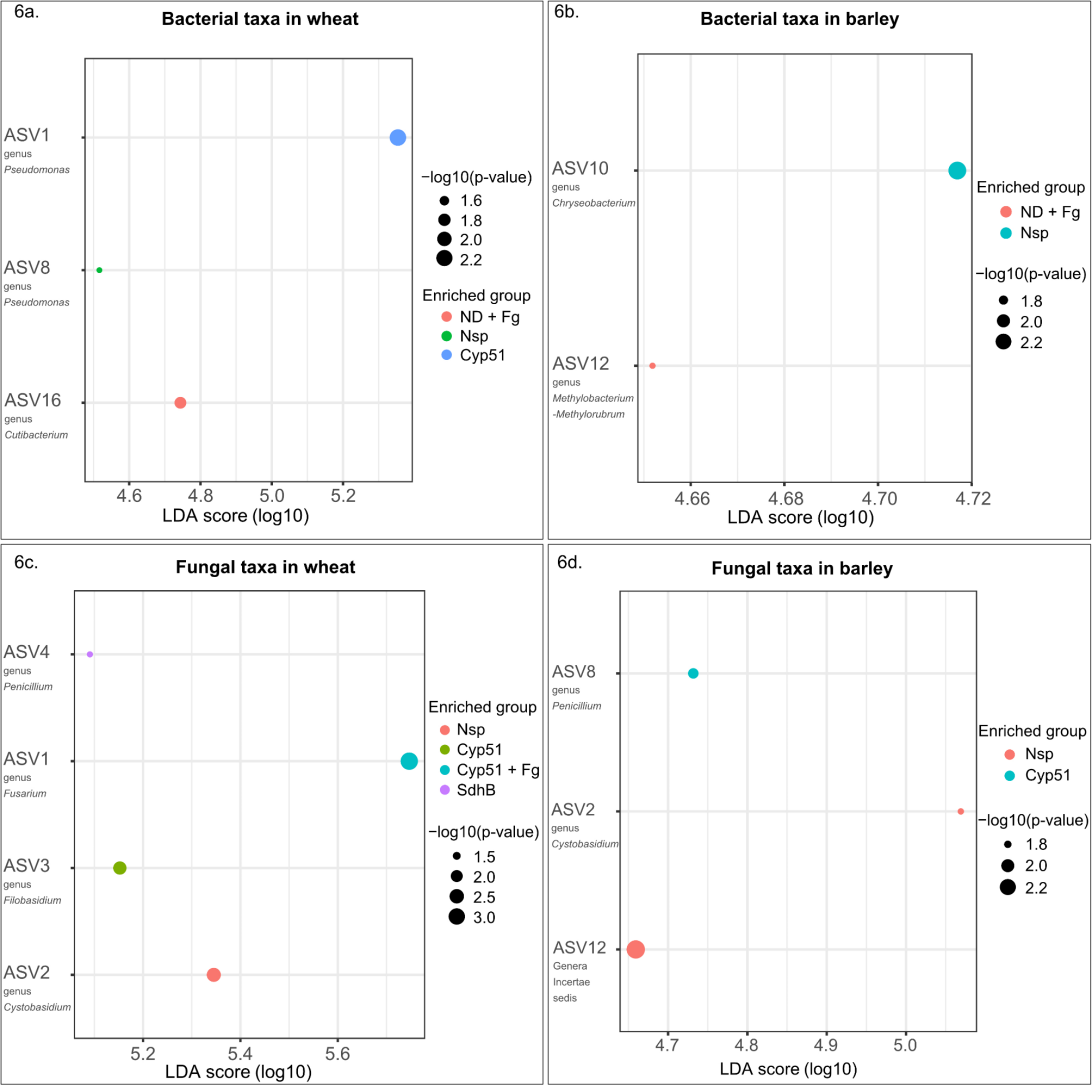
Linear discriminant analysis Effect Size (LEfSe) plots representing the bacterial and fungal ASVs distinguishing the different treatments in wheat and barley. Plots **a** and **b** represent bacterial ASVs, while **c** and **d** represent the fungal ASVs. Linear discriminatory (LDA) score and p-value cut-offs are > = 3.0 and < 0.05, respectively

Linear discriminant analysis Effect Size (LEfSe) was used to identify differentially abundant ASVs in each treatment (Fig. 6a-d). The discriminatory value or LDA score was used to evaluate the extent to which individual ASVs could be used to distinguish treatments. A cut-off of LDA score > = 3.0 and a p-value threshold of *p* < 0.05 was used to identify the ASVs characteristic of each treatment, and the results of the analysis were plotted in a dot plot format. Three bacterial and four fungal ASVs exhibited differential abundance between treatment groups in wheat (Fig. 6a, c), while two bacterial and three fungal ASVs were differentially abundant in barley (Fig. 6b, d).

The differentially abundant bacterial ASVs in wheat included ASV16 (*Cutibacterium*) in ND + Fg samples, ASV8 (*Pseudomonas*) in Nsp samples and ASV1 (*Pseudomonas*) in Cyp51 samples (Fig. 6a). Similarly, the differentially abundant bacterial ASVs in barley included ASV12 (*Methylobacterium-Methylorubrum*) in ND + Fg samples and ASV10 (*Chryseobacterium*) in Nsp samples (Fig. 6b). Among the fungal communities in wheat, ASV2 (*Cystobasidium*) was differentially abundant in the Nsp samples (Nsp), ASV3 (*Filobasidium*) in Cyp51 samples, ASV1 (*Fusarium*) in Cyp51 + Fg samples and ASV4 (*Penicillium*) in SdhB samples (Fig. 6c). In barley, ASV2 (*Cystobasidium*) and ASV12 (undefined genus) were differentially abundant in the Nsp samples while ASV8 (*Penicillium*) defined the Cyp51 samples (Fig. 6d).

### *F. graminearum* infection alters bacterial and fungal co-occurrence patterns in leaves sprayed with double-stranded RNA

For microbial co-occurrence network analysis, ninety-four (48 from wheat and 46 from barley) of the 126 samples sequenced, belonging the dsRNA (Nsp, Cyp51, SdhB) and dsRNA + Fg (Nsp + Fg, Cyp51 + Fg, SdhB + Fg) treatment groups were utilized for bacterial and fungal network construction.

**Fig. 7:**
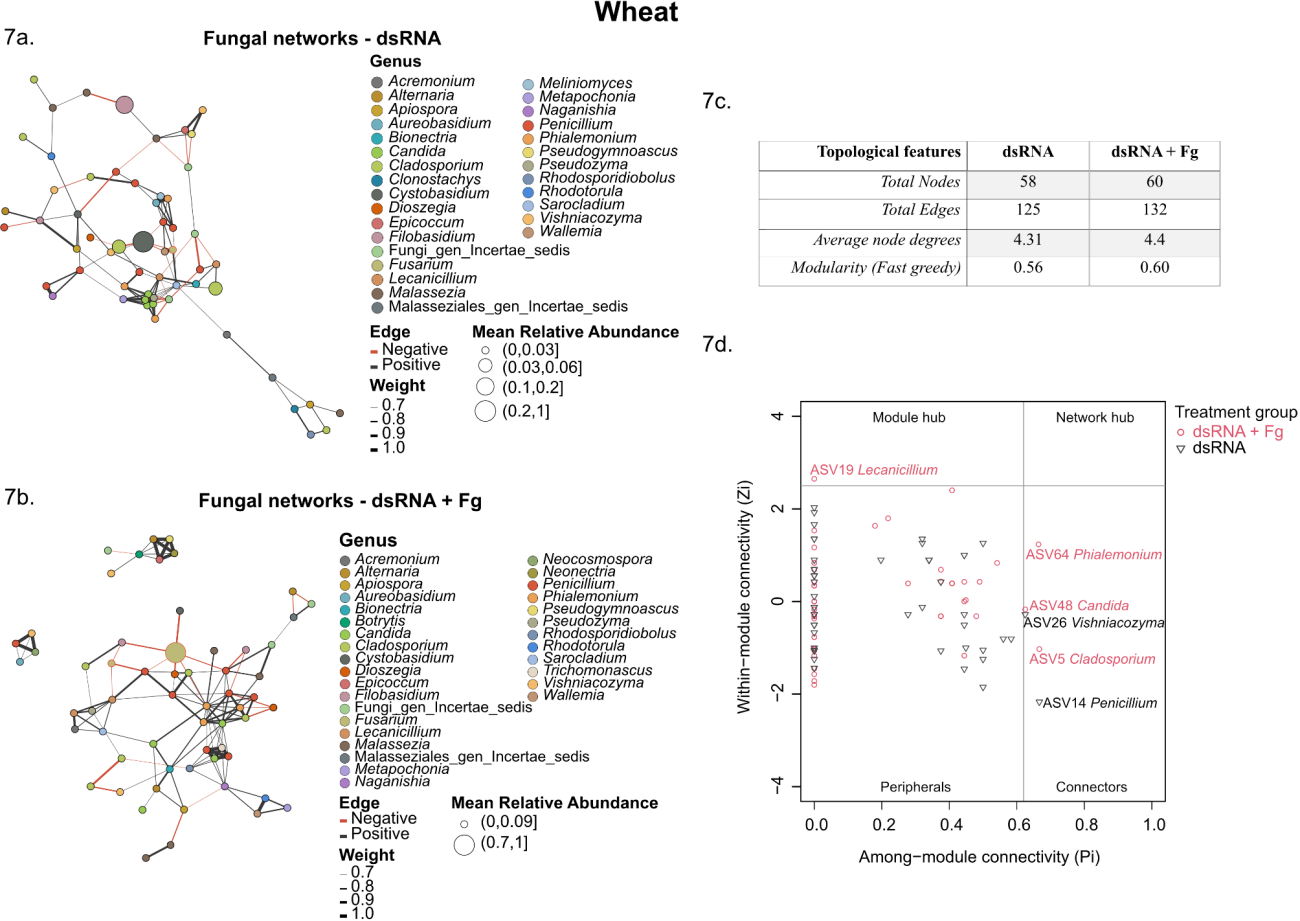
Genus-level fungal co-occurrence networks in dsRNA-treated wheat. The ASVs are represented as nodes. The size of each node is proportional to the relative abundance of the corresponding ASV. Nodes belonging to the genus Fusarium are highlighted with red borders. The connections denote a strong and significant correlation (*r* > 0.8, *P* < 0.05). Black lines or edges indicate positive interactions and red lines or edges indicate negative interactions. The thickness of the lines are proportional to the weight. Panels **a** and **b** show fungal co-occurrence networks in the dsRNA and dsRNA + Fg treatments in wheat, respectively. Panel **c** summarizes the main topological features observed in the aforementioned networks. Panel **d** shows the ZiPi plot for the fungal ASVs in wheat, revealing the importance of the different ASVs within and among modules in the network. A cut-off of Zi = 2.5 and Pi = 0.62 was used to distinguish the different roles. The genera *Penicillium*, *Vishniacozyma*, *Cladosporium*, *Candida* and *Phialemonium* were identified as connectors while *Lecanicillium* was identified as a module hub

**Fig. 8:**
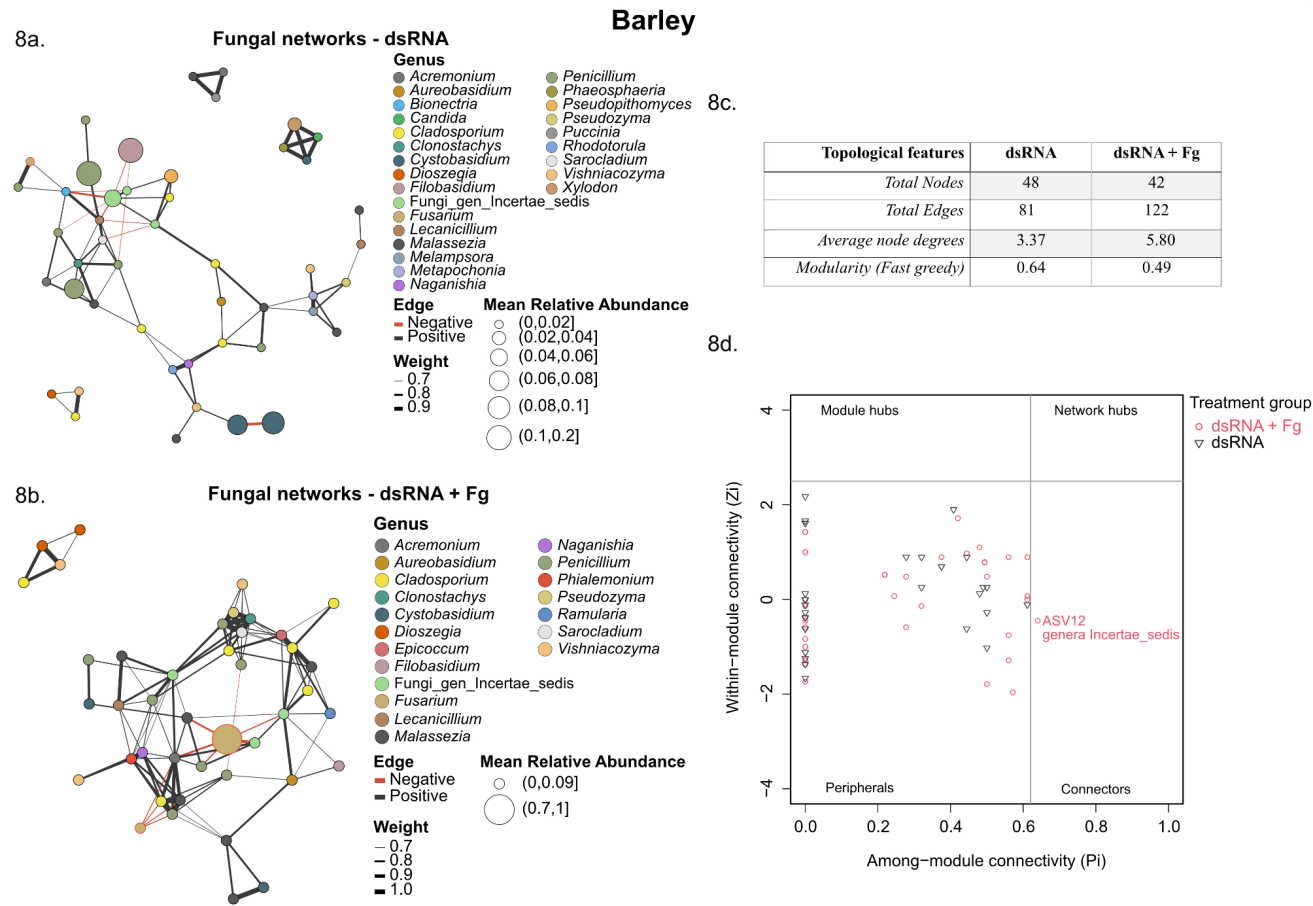
Genus-level fungal co-occurrence networks in dsRNA-treated barley. The ASVs are represented as nodes. The size of each node is proportional to the relative abundance of the corresponding ASV. Nodes belonging to the genus Fusarium are highlighted with red borders. The connections denote a strong and significant correlation (*r* > 0.8, *P* < 0.05). Black lines or edges indicate positive interactions and red lines or edges indicate negative interactions. The thickness of the lines are proportional to the weight. Panels **a** and **b** show fungal co-occurrence networks in the dsRNA and dsRNA + Fg treatments in barley, respectively. Panel **c** summarizes the main topological features observed in the aforementioned networks. Panel **d** shows the ZiPi plot for the fungal ASVs in barley, revealing the importance of the different ASVs within and among modules in the network. Scores of Zi = 2.5 and Pi = 0.62 were used to distinguish the different roles. An undefined genus was identified as a connector

At the genus level, smaller and sparse network clusters with strong internal relationships were observed for bacterial communities in wheat and barley (Additional file 2: Fig. S5a-d). In addition, the relationships detected between the bacterial ASVs were mostly positive but also included negative relations (*r* = 0.8, *p* < 0.05). The dsRNA-treated group had 27 bacterial nodes in wheat and 29 bacterial nodes in barley (Additional file 11: Table S10), but upon inoculation with *F. graminearum* (dsRNA + Fg), the number of bacterial nodes stayed relatively the same in wheat (26), while it increased in barley (75) (Additional file 11: Table S10). Contrary to observations in the bacterial networks, dense clusters were observed for the fungal communities in both hosts. In addition, both positive and negative interactions were observed between the fungal ASVs. The number of fungal nodes increased from 58 to 60 upon *F. graminearum* inoculation in wheat (Fig. 7a, b) but decreased from 48 to 42 in barley (Fig. 8a, b). Overall, inoculation with *F. graminearum* lowered bacterial community interactions and increased fungal interactions in dsRNA-sprayed wheat and barley leaves, as evident from the changes in the number of edges between the groups (Figs. 7c and 8c). The co-occurrence patterns were further characterized by computing average node degree and modularity (Figs. 7c and 8c) (Additional file 12: Table S11). Inoculation with *F. graminearum* increased the modularity of both bacterial and fungal networks in dsRNA-sprayed wheat leaves, whereas it increased bacterial modularity and lowered fungal modularity in dsRNA-treated barley leaves. Additional topological features from the bacterial and fungal networks are catalogued in supplementary file (Additional file 12: Table S11).

The Zi and Pi scores were then computed to evaluate the significance of each node in the network and categorize identified ASVs into the roles of peripherals, connectors, module hubs and network hubs, thereby revealing potential key taxa. All the nodes from the bacterial networks in wheat and barley were categorized as peripherals (Zi < 2.5 and Pi < 0.62), revealing that the identified nodes are only connected to other nodes within their own modules and thus do not play a significant role in maintaining the bacterial networks upon dsRNA spray (Additional file 2: Fig. S6). The fungal nodes classified from the co-occurrence patterns revealed mostly peripherals, a few connectors and one module hub. In wheat, ASV14 (*Penicillium*) and ASV26 (*Vishniacozyma*) from the dsRNA group and ASV5 (*Cladosporium*), ASV48 (*Candida*) and ASV64 (*Phialemonium*) from the dsRNA + Fg group were identified as connectors (Zi < 2.5 and Pi > 0.62), while ASV19 from the dsRNA + Fg group and assigned to the genus *Lecanicillium* was identified as a module hub (Zi > 2.5 and Pi < 0.62) (Fig. 7d). In barley, ASV12 (undefined genus) was identified as a connector from the dsRNA + Fg group (Fig. 8d).

## Discussion

The microbial communities of the phyllosphere are predominated by bacteria [75]. Our findings also support this conclusion since more bacterial than fungal ASVs were identified by sequencing. For all eight treatments examined, the most abundant bacterial phyla in the phyllosphere were *Proteobacteria*, *Actinobacteria*, *Bacteroidota* and *Firmicutes*. This is consistent with previous studies on bacterial communities in wheat leaves [76, 77]. Other studies have also shown the dominance of these microbial taxa in the phyllosphere and other plant organs of various crops and native plants, although the relative abundance of individual taxa may vary depending on host genotype, human intervention, and geographic location [78–80]. The genus *Pseudomonas*, which was identified across all treatments in wheat and barley, is ubiquitous in the phyllosphere [81].

Of the fungal phyla identified in the eight treatments, most belonged to *Ascomycota* and *Basidiomycota*. The fungal communities in the phyllosphere exhibit high species diversity and contribute to plant growth and metabolism via complex relationships [82, 83]. They also play essential roles in driving carbon and nitrogen cycling in agronomic crops and forest environments [84, 85]. Taxa identified in this study such as *Cladosporium* sp., *Alternaria* sp., *Dioszegia* sp. and *Vishniacozyma* sp. have previously been identified as integral parts of the wheat and barley phyllosphere mycobiome [76, 86–88]. Species from the genera *Filobasidium* and *Cystobasidium*, which were prevalent in all treatments in both wheat and barley, have previously been identified in wheat flag leaf and leaf samples [76, 89] .

### Spraying dsRNA differentially affects bacterial communities while maintaining fungal diversity and composition in wheat and barley

The Shannon diversity measures for bacterial communities in both wheat and barley were similar in all treatments except SdhB and SdhB + Fg, which displayed lower diversity. Beta diversity plots also revealed separate clustering of bacterial communities from SdhB samples in wheat. In barley though, the different control and dsRNA treatments clustered separately, with an overlap only between dsRNA Cyp51- and dsRNA SdhB- sprayed samples. These PCoA plots based on the Bray-Curtis distance therefore revealed dissimilarities in the composition of bacterial communities between the different treatments. In addition, dsRNA-specific and host-specific differences were also identified. Composition plots showed no major changes in the composition of the top 20 bacterial genera in wheat. However, the relative abundance of the candidates from the top 20 genera increased upon dsRNA spray. In barley, changes in the relative abundance varied depending on the dsRNA sprayed. In particular, the relative abundance of *Methylobacterium-methylorubrum* increased significantly in dsRNA Cyp51- sprayed samples, while *Acinetobacter* increased significantly in dsRNA SdhB- sprayed samples. However, genera such as *Pseudomonas*, *Sphingomonas*, *Cutibacterium* and *Methylobacterium-Methylorubrum* were found to be ubiquitous across all treatments and in both hosts, indicating that spraying dsRNA does not impair the survival/existence of bacteria ubiquitous to the wheat and barley phyllosphere. Interestingly, it was observed that the relative abundance of the genus *Pseudomonas* increased upon both dsRNA spray and *F. graminearum* inoculation in wheat, but not as much in barley. This difference could be attributed to the significance of this genus in shaping the native microbial communities in the specific cultivars of wheat and barley chosen in this study. Further analysis using meta-genomic and meta-transcriptomic approaches will help gain a deeper understanding of the genes and pathways that govern such intricate microbial community assemblies. Together, these results indicate that the effects of dsRNA on the diversity and structure of the bacterial communities of the phyllosphere varied depending on the gene targeted and the host studied.

The diversity, structure and composition of the fungal communities, on the other hand, were more uniform across both hosts. No obvious differences in the alpha- and beta- diversity measures were observed between the no dsRNA and dsRNA samples, indicating dsRNA spray did not impact the diversity of fungal communities in both wheat and barley. Studies have reported that high species richness and the presence of direct competitors can positively influence plant health, as other microorganisms compete for space and resources, increasing competition for the pathogen as a result [90, 91]. In addition, the composition plots and heat maps revealed the ubiquitous presence of highly abundant fungal genera like *Cladosporium*, *Cystobasidium*, *Filobasidium* and *Penicillium* in both the no dsRNA and dsRNA samples. This similarity in fungal composition could be attributed to the fungal communities being more stable and displaying resistance in response to disturbance (dsRNA spray) in their environment [92]. These observations together underpin that spraying dsRNA does not alter the native fungal communities of the phyllosphere in wheat and barley.

### *F. graminearum* inoculation alters fungal co-occurrence patterns in dsRNA-sprayed plants

Network topology analyses can reveal important network nodes and edges while also facilitating comparisons between networks. In these analyses, the node degree indicates the number of direct connections for a specific ASV, the closeness centrality value indicates how quickly information spreads from a given node to other reachable nodes, and the betweenness centrality of a node reflects the effects of one microbe on the co-occurrence of other nodes [93]. In addition, the modularity may reflect biotic interactions between closely associated ASVs in an ecological community [94]. Our results showed that the bacterial and fungal networks for all of the studied treatments had comparable degrees, eigenvectors, and closeness centralities, indicating stable and uninterrupted networks. Network topology analyses revealed that the modularity of the fungal networks in the dsRNA treatments was comparatively higher or not appreciably different than the dsRNA + Fg treatments. This suggests that dsRNA provided a range of ecological niches to allow a greater diversity of fungi to flourish, whereas *F. graminearum* inoculation reduced the range of these available niches. Conversely, the modularity of the bacterial networks in the dsRNA + Fg treatments was greater than the dsRNA only treatments in both hosts. In addition, all of the bacterial modules were highly connected within themselves, while the different modules remained isolated from each other.

Bacteria and fungi identified through other analyses as defining the microbial communities of dsRNA-sprayed wheat and barley leaves were also represented in the co-occurrence patterns. Interactions within the bacterial communities were mostly positive, while there was a mix of both positive and negative interactions between fungal communities, with a noticeable increase in negative interactions upon inoculation with *F*. *graminearum*. This reveals that *F. graminearum* establishes itself within the fungal community by interacting negatively and reducing the relative abundances of the top genera.

Identifying microbial keystone or hub taxa is extremely valuable for the sustainable development of cereal ecosystems as they play vital roles in helping other microbes to maintain the dynamics of microbial networks [95]. Their importance is such that their disappearance can cause network collapse [96]. Co-occurrence network analyses were therefore performed to evaluate the complexity of the targeted microbiota [97], which revealed that the numbers of nodes, edges, and modules in both the bacterial and fungal networks were sensitive to both the host plant species and infection by the pathogen *F. graminearum*. It is important to note that microbial co-occurrence analyses do not always predict exact real-time networks and therefore require further omics- and culture-based strategies to obtain deeper insights into relationships within microbial communities.

The results of the analyses described above collectively indicate that foliar spraying with dsRNA has varied effects on the bacterial communities and negligible effects on the fungal communities of the phyllosphere. Previous studies on the microbiome have examined the phyllosphere and flag leaf samples in wheat [76, 77, 87, 89], and the phyllosphere fungal endophytes [88], the rhizosphere and grains [98, 99] in barley. This work further expands our understanding of plant microbial communities by characterizing those found in the barley and wheat phyllosphere. The number of samples per treatment in our experiments was limited because our study was greenhouse-based, so it would be desirable to conduct follow-up field studies to obtain additional insights into the effects of dsRNA on host microbial communities. Additionally, previous studies have shown that plant genotype and environmental conditions can have a considerable influence on the phyllosphere microbial communities [41, 75, 86]. Testing the effects of dsRNA spraying on the phyllosphere microbiota of different host cultivars and under varying environmental conditions could validate the results presented here and reveal potential genotype-specific effects. Overall, this pilot study shows that although rarer and less abundant ASVs change upon dsRNA spray, the ubiquitous bacterial and fungal components of the phyllosphere in wheat and barley remain unchanged.

## Conclusion

Spray-induced gene silencing (SIGS) is attracting considerable interest as a plant protection strategy because it has the potential to be an efficient and environmentally friendly alternative to conventional chemical fungicides and transgenic crops. Studies on several agricultural and horticultural crops have proven SIGS effective against diverse plant pathogens and pests. However, despite its proven efficiency in reducing the incidence and severity of plant diseases, several aspects of SIGS require further study to make it a practical plant protection strategy. Leaves represent a large surface area of the plant and can act as entry points for pathogens and other microbes [100]. Moreover, the aerial parts of plants also influence growth, fitness and yield. Therefore, an important aspect of spraying dsRNA is its effect on the microbial communities, particularly in the phyllosphere. Our results address this need by providing novel insights into the effects of SIGS on the phyllosphere microbiome in wheat and barley. Using amplicon sequencing, we have shown that the diversity, structure and composition of the phyllosphere bacterial communities are subject to subtle changes upon exogenous dsRNA application, while the fungal communities remain largely unaffected. We also show that dsRNA does not impact the fungal compositional changes induced by *F. graminearum* inoculation in wheat and barley leaves. Further validation of these results through large-scale field studies can help incorporate how host genotype and environmental conditions influence the effect of dsRNA on phyllosphere communities, and reinforce the safety of SIGS for practical use.

## Electronic supplementary material

Below is the link to the electronic supplementary material.

Supplementary Material 1

Supplementary Material 2

Supplementary Material 3

Supplementary Material 4

Supplementary Material 5

Supplementary Material 6

Supplementary Material 7

Supplementary Material 8

Supplementary Material 9

Supplementary Material 10

Supplementary Material 11

Supplementary Material 12

## Data Availability

The data that support the study are in the article and supplementary materials. Raw sequences have been deposited at the National Centre for Biotechnology Information (NCBI) Sequence Read Archive (SRA) under the BioProject accession PRJNA980286. All code created during this work can be obtained from the following GitHub repository: https://github.com/samratencode/amplicon_analysis.git.
