# Supplementary material for: The impact of spray-induced gene silencing on cereal phyllosphere microbiota
Source: Environ Microbiome. 2025 Jan 8;20:1. doi: 10.1186/s40793-024-00660-8 (PMC11716504; doi:10.1186/s40793-024-00660-8)
Supplement: Supplementary file 2 — Supplementary Material 2 [file 40793_2024_660_MOESM2_ESM.pdf]

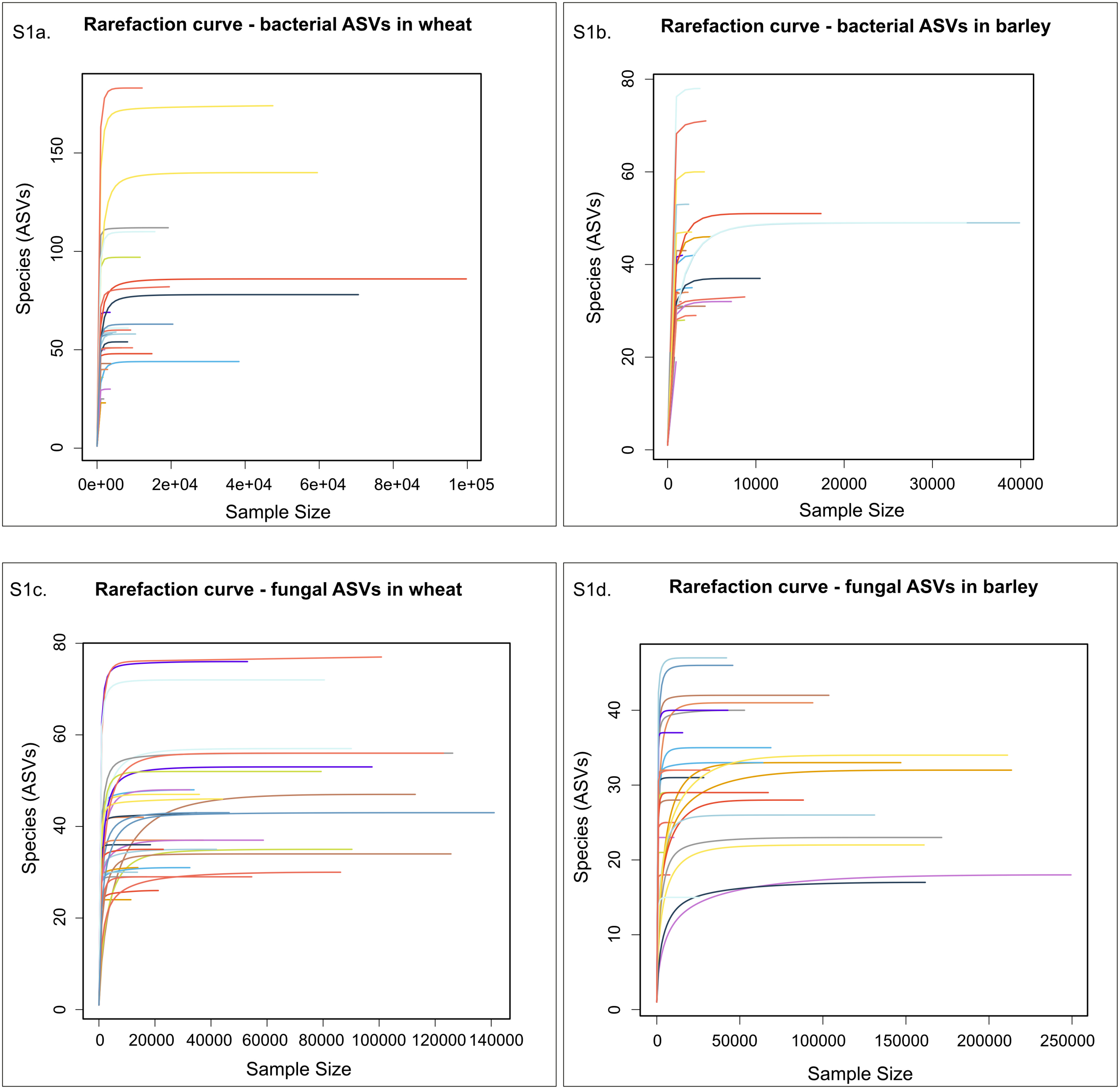
S1a.
Rarefaction curve - bacterial ASVs in wheat
Species (ASVs)
0
50
100
150
0e+00
2e+04
4e+04
6e+04
8e+04
1e+05
Sample Size
S1b.
Rarefaction curve - bacterial ASVs in barley
Species (ASVs)
0
20
40
60
80
0
10000
20000
30000
40000
Sample Size
S1c.
Rarefaction curve - fungal ASVs in wheat
Species (ASVs)
0
20
40
60
80
0
20000
40000
60000
80000
100000
120000
140000
Sample Size
S1d.
Rarefaction curve - fungal ASVs in barley
Species (ASVs)
0
10
20
30
40
0
50000
100000
150000
200000
250000
Sample Size

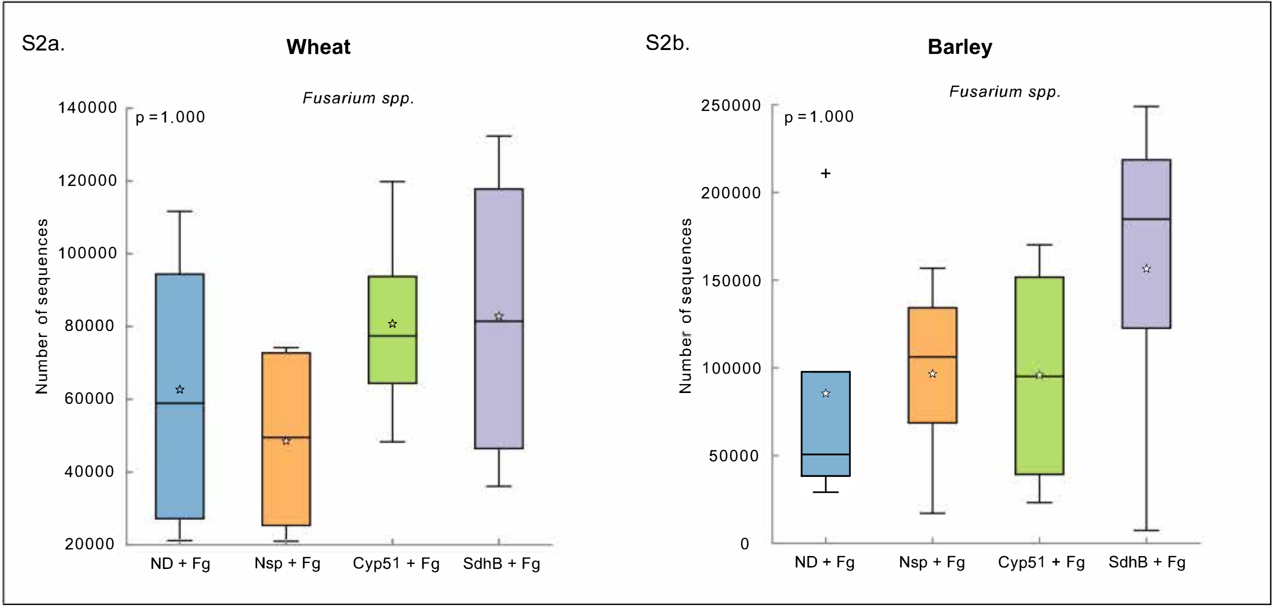
S2a.
Wheat
Fusarium spp.
p = 1.000
Number of sequences
140000
120000
100000
80000
60000
40000
20000
ND + Fg
Nsp + Fg
Cyp51 + Fg
SdhB + Fg
S2b.
Barley
Fusarium spp.
p = 1.000
Number of sequences
250000
200000
150000
100000
50000
0
ND + Fg
Nsp + Fg
Cyp51 + Fg
SdhB + Fg

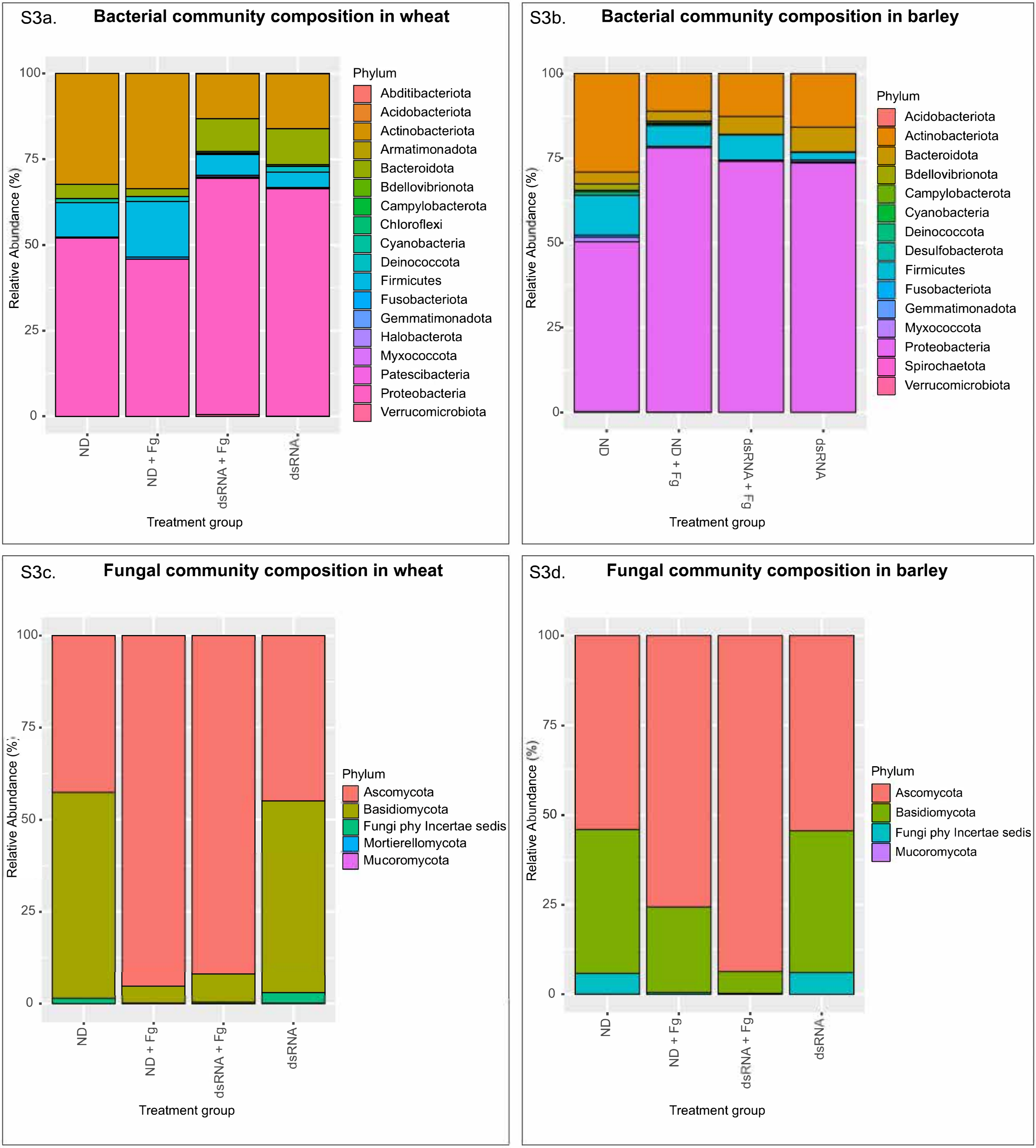
S3a.
Bacterial community composition in wheat
Relative Abundance (%)
0
25
50
75
100
ND
ND + Fg
dsRNA + Fg
dsRNA
Treatment group
Phylum
Abditibacteriota
Acidobacteriota
Actinobacteriota
Armatimonadota
Bacteroidota
Bdellovibrionota
Campylobacterota
Chloroflexi
Cyanobacteria
Deinococcota
Firmicutes
Fusobacteriota
Gemmatimonadota
Halobacterota
Myxococcota
Patescibacteria
Proteobacteria
Verrucomicrobiota
S3b.
Bacterial community composition in barley
Relative Abundance (%)
0
25
50
75
100
ND
ND + Fg
dsRNA + Fg
dsRNA
Treatment group
Phylum
Acidobacteriota
Actinobacteriota
Bacteroidota
Bdellovibrionota
Campylobacterota
Cyanobacteria
Deinococcota
Desulfobacterota
Firmicutes
Fusobacteriota
Gemmatimonadota
Myxococcota
Proteobacteria
Spirochaetota
Verrucomicrobiota
S3c.
Fungal community composition in wheat
Relative Abundance (%)
0
25
50
75
100
ND
ND + Fg
dsRNA + Fg
dsRNA
Treatment group
Phylum
Ascomycota
Basidiomycota
Fungi phy Incertae sedis
Mortierellomycota
Mucoromycota
S3d.
Fungal community composition in barley
Relative Abundance (%)
0
25
50
75
100
ND
ND + Fg
dsRNA + Fg
dsRNA
Treatment group
Phylum
Ascomycota
Basidiomycota
Fungi phy Incertae sedis
Mucoromycota

S4a. **Core prevalence - bacterial taxa in wheat**

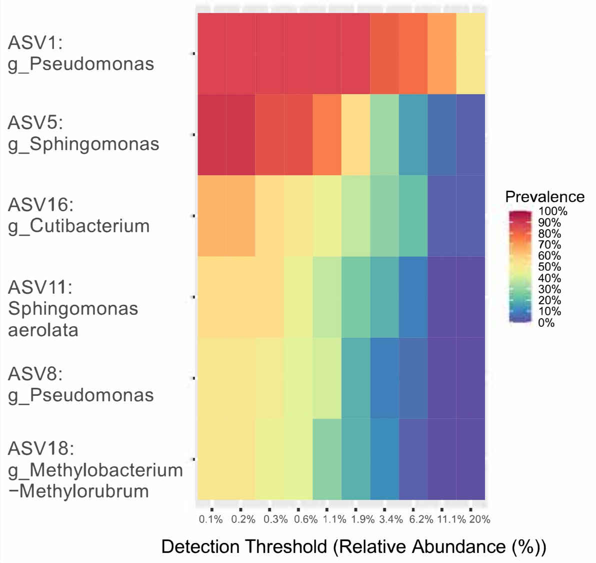

S4b. **Core prevalence - bacterial taxa in barley**

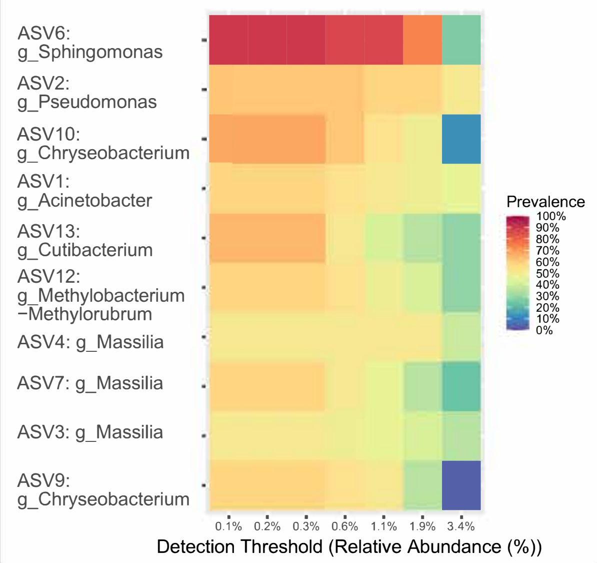

S4c. **Core prevalence - fungal taxa in wheat**

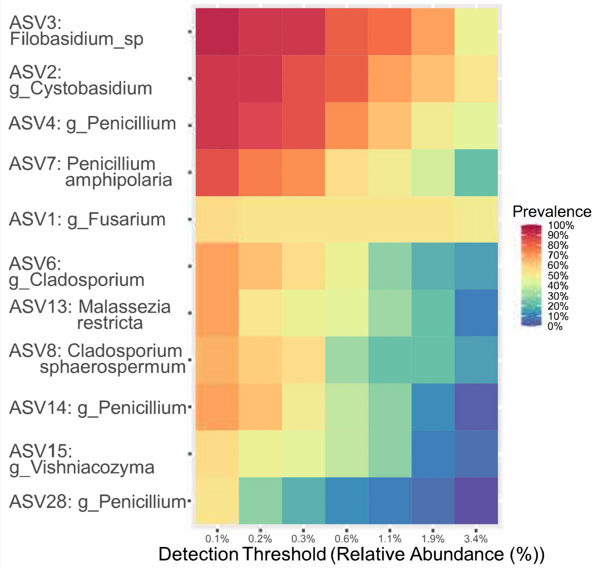

S4d. **Core prevalence - fungal taxa in barley**

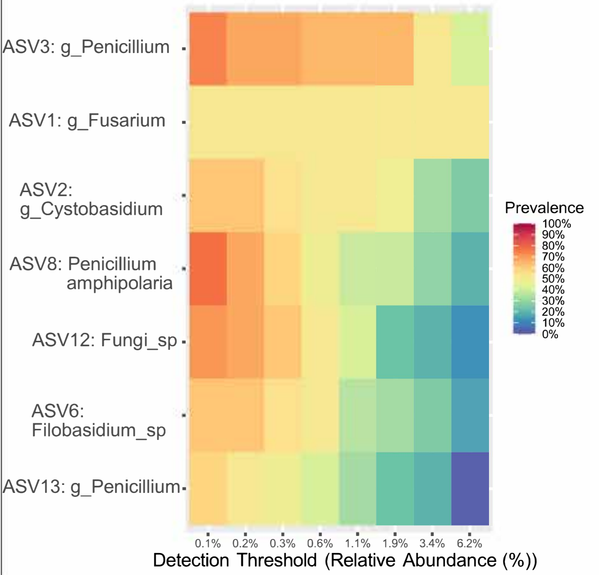

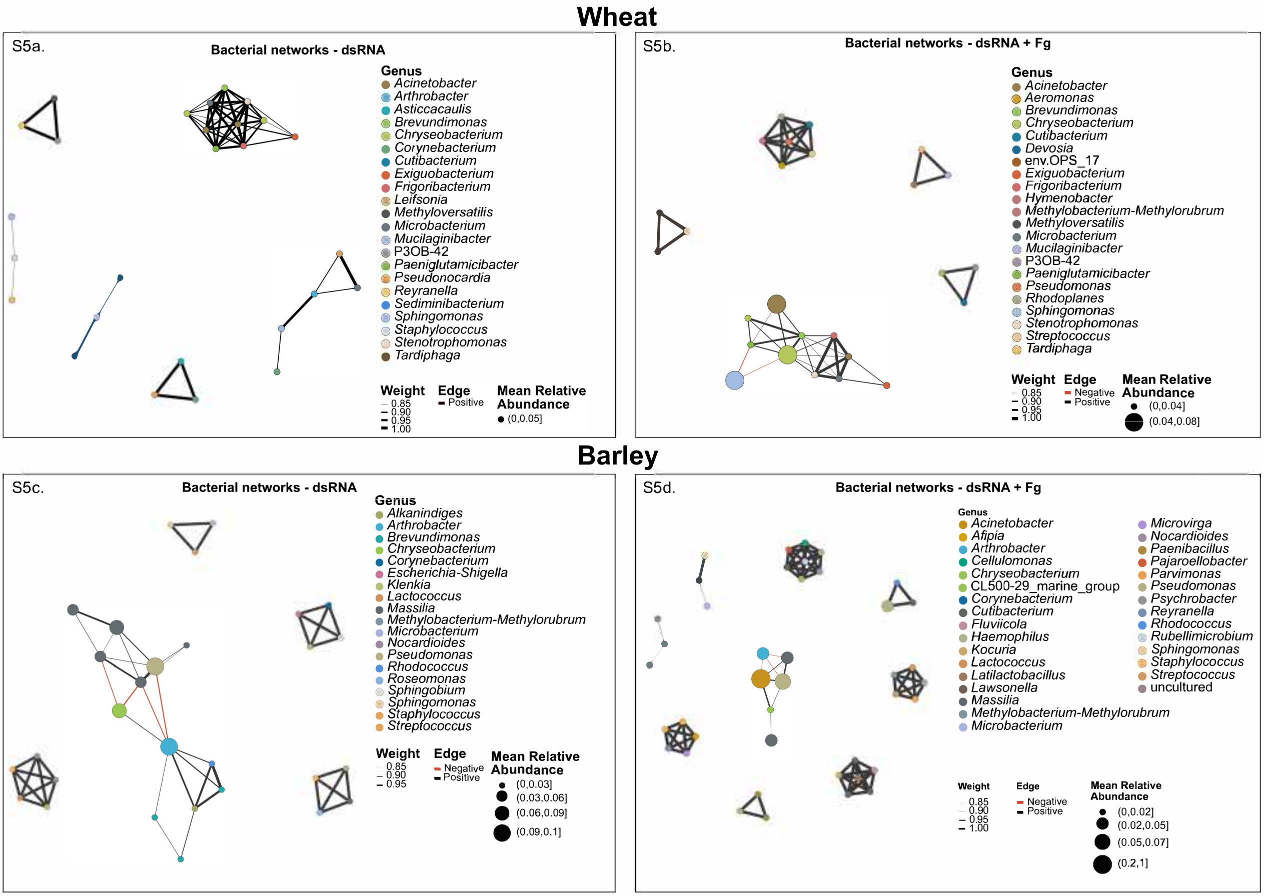

Wheat
S5a.
Bacterial networks - dsRNA
Genus
Acinetobacter
Arthrobacter
Asticcacaulis
Brevundimonas
Chryseobacterium
Corynebacterium
Cutibacterium
Exiguobacterium
Frigoribacterium
Leifsonia
Methyloversatilis
Microbacterium
Mucilaginibacter
P3OB-42
Paeniglutamicibacter
Pseudonocardia
Reyranella
Sediminibacterium
Sphingomonas
Staphylococcus
Stenotrophomonas
Tardiphaga
Weight
0.85
0.90
0.95
1.00
Edge
Positive
Mean Relative Abundance
(0,0.05]
S5b.
Bacterial networks - dsRNA + Fg
Genus
Acinetobacter
Aeromonas
Brevundimonas
Chryseobacterium
Cutibacterium
Devosia
env.OPS_17
Exiguobacterium
Frigoribacterium
Hymenobacter
Methylobacterium-Methylorubrum
Methyloversatilis
Microbacterium
Mucilaginibacter
P3OB-42
Paeniglutamicibacter
Pseudomonas
Rhodoplanes
Sphingomonas
Stenotrophomonas
Streptococcus
Tardiphaga
Weight
0.85
0.90
0.95
1.00
Edge
Negative
Positive
Mean Relative Abundance
(0,0.04]
(0.04,0.08]
Barley
S5c.
Bacterial networks - dsRNA
Genus
Alkanindiges
Arthrobacter
Brevundimonas
Chryseobacterium
Corynebacterium
Escherichia-Shigella
Klenkia
Lactococcus
Massilia
Methylobacterium-Methylorubrum
Microbacterium
Nocardioides
Pseudomonas
Rhodococcus
Roseomonas
Sphingobium
Sphingomonas
Staphylococcus
Streptococcus
Weight
0.85
0.90
0.95
Edge
Negative
Positive
Mean Relative Abundance
(0,0.03]
(0.03,0.06]
(0.06,0.09]
(0.09,0.1]
S5d.
Bacterial networks - dsRNA + Fg
Genus
Acinetobacter
Afipia
Arthrobacter
Cellulomonas
Chryseobacterium
CL500-29_marine_group
Corynebacterium
Cutibacterium
Fluviicola
Haemophilus
Kocuria
Lactococcus
Latilactobacillus
Lawsonella
Massilia
Methylobacterium-Methylorubrum
Microbacterium
Microvirga
Nocardioides
Paenibacillus
Pajaroellobacter
Parvimonas
Pseudomonas
Psychrobacter
Reyranella
Rhodococcus
Rubellimicrobium
Sphingomonas
Staphylococcus
Streptococcus
uncultured
Weight
0.85
0.90
0.95
1.00
Edge
Negative
Positive
Mean Relative Abundance
(0,0.02]
(0.02,0.05]
(0.05,0.07]
(0.2,1]

S6a.

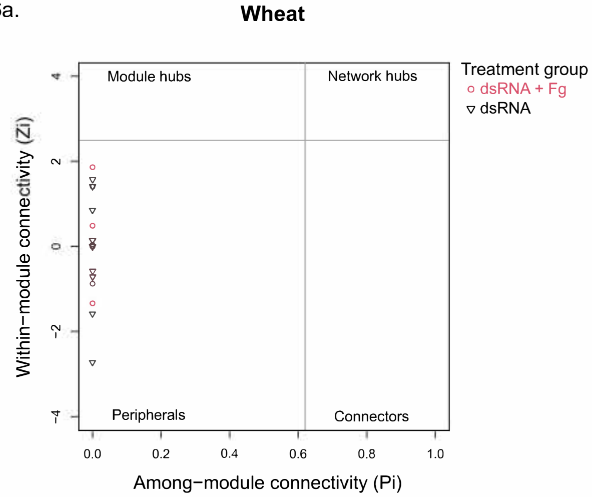

S6b.

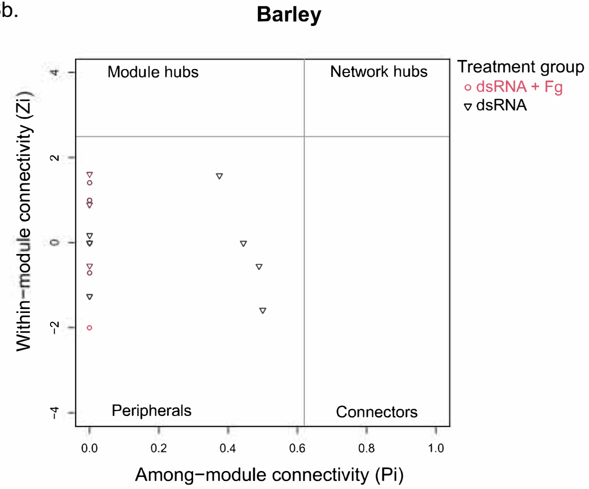

S6c.

Topological features	dsRNA	dsRNA + Fg
Total Nodes	27	26
Total Edges	54	51
Average node degrees	4.0	3.92
Modularity (Fast greedy)	0.46	0.62

S6d.

Topological features	dsRNA	dsRNA + Fg
Total Nodes	29	75
Total Edges	49	41
Average node degrees	3.37	3.65
Modularity (Fast greedy)	0.70	0.82
